# OX40 and 4-1BB delineate distinct immune profiles in sarcoma

**DOI:** 10.1080/2162402X.2022.2066050

**Published:** 2022-05-09

**Authors:** MJ Melake, HG Smith, D Mansfield, E Davies, MT Dillon, AC Wilkins, EC Patin, M Pedersen, R Buus, AA Melcher, K Thway, AB Miah, SH Zaidi, AJ Hayes, TR Fenton, KJ Harrington, M McLaughlin

**Affiliations:** a Targeted Therapy Team, The Institute of Cancer Research, London, UK; b Digestive Disease Center, Bispebjerg and Frederiksberg Hospital, University of Copenhagen, Denmark; c Translational Immunotherapy Team, The Institute of Cancer Research, London, UK; d The Royal Marsden Hospital, London, UK; e The Breast Cancer Now Toby Robins Research Centre, The Institute of Cancer Research, London, UK; f University of Southampton, Somers Cancer Research Building MP824, Southampton General Hospital, Southampton, UK

**Keywords:** TNFSFR4, CD137, TNFRSF9, agonist, immunotherapy

## Abstract

Systemic relapse after radiotherapy and surgery is the major cause of disease-related mortality in sarcoma patients. Combining radiotherapy and immunotherapy is under investigation as a means to improve response rates. However, the immune contexture of sarcoma is understudied. Here, we use a retrospective cohort of sarcoma patients, treated with neoadjuvant radiotherapy, and TCGA data. We explore therapeutic targets of relevance to sarcoma, using genomics and multispectral immunohistochemistry to provide insights into the tumor immune microenvironment across sarcoma subtypes. Differential gene expression between radioresponsive myxoid liposarcoma (MLPS) and more radioresistant undifferentiated pleomorphic sarcoma (UPS) indicated UPS contained higher transcript levels of a number of immunotherapy targets (CD73/*NT5E*, CD39/*ENTPD1*, CD25/*IL2RA*, and 4–1BB/*TNFRSF9*). We focused on 4–1BB/*TNFRSF9* and other costimulatory molecules. In TCGA data, 4–1BB correlated to an inflamed and exhausted phenotype. OX40/*TNFRSF4* and 4–1BB/*TNFRSF9* were highly expressed in sarcoma subtypes versus other cancers. Despite OX40 and 4–1BB being described as Treg markers, we identified that they delineate distinct tumor immune profiles. This was true for sarcoma and other cancers. While only a limited number of samples could be analyzed, spatial analysis of OX40 expression identified two diverse phenotypes of OX40+ Tregs, one associated with and one independent of tertiary lymphoid structures (TLSs). Patient stratification is of intense interest for immunotherapies. We provide data supporting the viewpoint that a cohort of sarcoma patients, appropriately selected, are promising candidates for immunotherapies. Spatial profiling of OX40+ Tregs, in relation to TLSs, could be an additional metric to improve future patient stratification.

Soft-tissue sarcomas (STS) comprise a heterogeneous group of rare cancers derived from mesenchymal tissue and account for 1% of all adult cancers.^1^ Although capable of occurring at virtually any anatomical site, STS most commonly occur in the limbs and limb girdles, where they are often referred to as extremity soft-tissue sarcomas (ESTS).^2^ Surgery and radiotherapy, delivered pre- or post-operatively in patients at high risk of relapse, remains the cornerstone of management for patients presenting with primary localized ESTS.^3^ Systemic relapses occur in up to a third of patients, accounting for the majority of disease-related mortality.^2,4^ Despite an ability to predict patients at increased risk of systemic relapse, prevention and/or treatment of metastatic sarcoma remains an area of significant unmet need.^5^

Immune checkpoint inhibitors (ICIs) targeting the PD-1 (programmed cell death protein 1) and CTLA-4 (cytotoxic T-lymphocyte-associated protein 4) axes have revolutionized the management of patients with historically poor prognoses.^6^ However, the initial results from clinical trials with ICIs in patients with metastatic sarcoma were disappointing, with poor response rates in many subtypes.^7–10^ One proffered explanation for this poor response is that sarcomas are ‘immune-cold’ in comparison with more responsive pathologies.^11–13^ Sarcomas are not a single disease entity but, rather, a group of cancers with diverse biologies and, consequently, diverse responses to therapy. Recent trial results indicate patients with undifferentiated pleomorphic sarcomas (UPS) may be more responsive to anti-PD-1 therapy than patients with other sarcoma subtypes.^11,14^ Not only is UPS the most common subtype amongst patients with ESTS, it is also associated with the highest rates of systemic relapse and poorest survival outcomes.^4^ As such, strategies to optimize the response to immunotherapies in this sarcoma subtype are of great interest.

In this study, we sought to profile the basal tumor immune environment of patients prior to treatment with radiotherapy. We initially, using a retrospective cohort of patients, sought to compare UPS,^13^ which is relatively more radioresistance compared to other sarcoma subtypes, to myxoid liposarcoma (MLPS), which is relatively more radiosensitive compared to other subtypes. Our objective was to identify immunotherapy targets in sarcoma patients with an increased probability of radioresistant disease, as well as increase the understanding of the immune contexture of sarcoma. Using both our retrospective cohort of clinical samples, and data from The Cancer Genome Atlas (TCGA), we identify *TNFRSF4*/OX40 and *TNFRSF9*/4-1BB as two immune markers of potential relevance to sarcoma. OX40 and 4–1BB are costimulatory receptors of the TNF receptor family, linked to enhanced survival, cytotoxicity, and shown to counteract T cell exhaustion.^15,16^ *TNFRSF4*/OX40 was highest in UPS, MFS (myxofibrosarcoma) and DDLS (dedifferentiated liposarcoma) compared to all other cancer types investigated. Despite previous publications linking *TNFRSF4*/OX40 and *TNFRSF9*/4-1BB to Tregs,^17,18^ we identified that these two transcripts could delineate distinct immune phenotypes. Additional spatial profiling indicated OX40+ Tregs could be further subdivided into tertiary lymphoid structure (TLS)-associated and TLS-independent Tregs.

## Methods

### Retrospective STS patient samples

Archival histopathological samples were retrieved from the Royal Marsden Hospital tissue bank. This study was approved by the institutional review board (Committee for Clinical Research No. CCR4852). Consent was confirmed for all patients. Pre-treatment biopsy specimens were sourced from archival blocks from patients who received neoadjuvant radiotherapy followed by surgical resection. To note, only pre-treatment biopsies are analyzed as part of this study. Archival tissue was retrieved from a further 2 UPS patients who did not receive pre-operative radiotherapy. Retrieved samples were limited to patients diagnosed with soft-tissue sarcoma of the extremities with the following histological subtypes – undifferentiated pleomorphic, myxofibrosarcoma, and myxoid liposarcoma. Samples from patients with recurrent or metastatic soft-tissue sarcomas were excluded.

### NanoString gene expression analysis

H&E sections from formalin-fixed paraffin embedded (FFPE) samples were outlined to guide macrodissection of viable areas of tumor by an expert soft-tissue sarcoma pathologist (KT). Macrodissection was performed on nuclear fast red-stained sections. RNA was extracted using the Allprep DNA/RNA FFPE kit (Qiagen) using the manufacturer’s protocol. Extracted RNA was quantified using a NanoDrop spectrophotometer (Thermo Fisher) and quality assessed on a BioAnalyzer 2100 (Agilent). 100 ng RNA was used for analysis using the Human nCounter PanCancer Immune Profiling Panel (NanoString Technologies). A customized 30-gene panel was included alongside the standard probe set. Custom probes were included against the following gene transcripts; *APEX1, APOBEC3B, ATR, BATF3, BRCA1, BRCA2, CFLAR, CLEC9A, DDB2, H2AFX, IFIT3, LIG4, CGAS, MDC1, MICA, MLH1, NBN, NLRP9, OAS1, OAS2, PARP1, PCNA, RAD51, RBBP8, RPA3, STK39, STING, TRADD, TREX1, XRCC4*. Normalization for hybridization and against housekeeping genes was performed using nSolver v4.0.70 (NanoString). Geometric mean of negative controls was chosen for background thresholding. Geometric mean of positive controls was used to compute normalization factor and lanes with a range outside 0.3–3 were flagged. One sample had to be excluded due to a normalization flag. This was a UPS sample with the highest 4–1BB level. Immune cell scoring from NanoString data was performed using the method described previously.^19^ Differentially expressed genes were determined in nSolver. Fold change cutoff of 2 is shown for transcripts significantly higher in UPS versus MLPS, with an FDR-corrected p-value of less than 0.05. Heatmaps were plotted using the ComplexHeatmap package. On log2 transformed data, hierarchical clustering was performed and k-means clustering of 2 is shown.

### Immune cell multiplex immunohistochemistry

FFPE tissue sections were stained for multiplex fluorescence-based IHC using opal reagents on 4 micrometre tissue sections (Akoya Biosciences, NEL811001KT). Sections were dewaxed and rehydrated. Sequential rounds of antigen retrieval, blocking, primary antibody incubation, wash, secondary-HRP conjugate, and opal reagent incubation were performed using the recommended manufacturers’ guidelines. Primary antibodies used were: mouse anti-human CD8 (Dako, clone M351501, 1:800, pH 9 retrieval), mouse anti-human CD68 (Dako, M087629, 1:750, pH 6 retrieval), mouse anti-human CD20 (Dako, M075529, 1:1000, pH 6 retrieval), mouse anti-human FOXP3 (Abcam, ab20034, 1:600, pH 6 retrieval), rabbit anti-human CD4 (Abcam, ab133616, 1:1000, pH 9 retrieval) and rabbit anti-human OX40 (Cell Signaling, 98785, 1:400, pH 6 retrieval). Completed slides were imaged using a Vectra 3 automated imaging platform (Akoya Biosciences) and resulting images unmixed using InForm v2.4.8 software (Akoya Biosciences). Tissue and cell detection, phenotyping, and spatial analysis was performed using Qupath v0.2.3, an open-source software for digital pathology image analysis.^20^

### TCGA sarcoma data analysis

Transcriptomic data from the TCGA were accessed using the R package cgdsr. TCGA sarcoma data were restricted to UPS, MFS, DDLS, and LMS (Leiomyosarcoma). LMS was excluded from analysis in Figure 4 due to dissimilarity to the other subtypes. We maintained the separation of UPS and MFS as distinct subtypes for our analyses. Although molecular data from the TCGA publication states that these two subtypes fall along a spectrum^13^ differing clinical outcomes to treatment act as a counterpoint to the pooling of these two subtypes.^4^ TCGA data for other cancer types were used as labeled and not subsetted. Where it was judged to aid the clarity of presentation, data were log2 transformed. Where this is the case, it is indicated. For survival probability analysis in Figure 2, 61 samples received radiotherapy (2 DDLS, 17 LMS, 17 MFS, 25 UPS), with 128 receiving no radiotherapy (32 DDLS, 68 LMS, 8 MFS, 20 UPS). Samples where radiotherapy status could not be clearly determined were excluded. Cancers in the TCGA dataset were selected for comparative purposes to sarcoma due to their responsiveness to immunotherapy, or those commonly referred to as immune-hot, immune-cold, or with low mutational burden.

**Figure 1.**
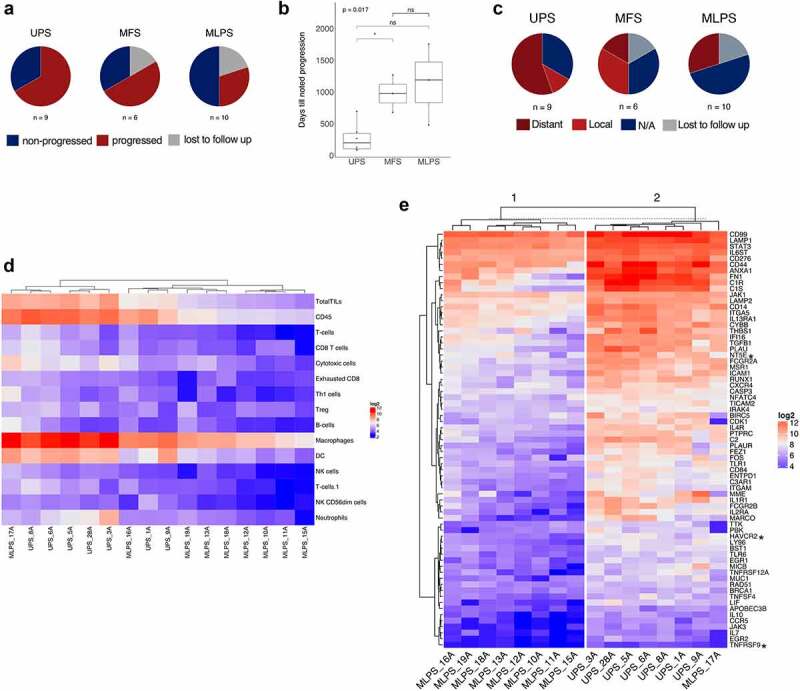
**Compared to MLPS, 4-1BB (*TNFRSF9*) and TIM3 (*HAVCR2*) are elevated in UPS patients**. (**a**) Progression shown by subtype after neoadjuvant radiotherapy and surgical resection. (**b**) Time from surgery until noted progression due to local recurrence or metastatic disease. Significance shown t-test, *p<0.05. UPS n=6, MFS n=3, MLPS n=3. (**c**) Breakdown of progression by distal, local relapse, no progression (NA), or unknown. (**d**) Non-hierarchical clustering analysis of immune cell abundance in MLPS and UPS samples derived from gene expression cell type scoring (Danaher et al., 2017) of NanoString pancancer immune panel data. (**e**) NanoString transcriptomics data showing differentially expressed genes between UPS and MLPS grouped by non-hierarchical clustering with samples split by k-means clustering into two clusters. Only significantly differentially expressed genes with an FDR adjusted p-value less than 0.05 and a fold change increase of 2 for UPS, relative to MLPS, are shown. *HAVCR2* (TIM-3), *NT5E* (CD73) and *TNFRSF9* (4-1BB/CD137) are indicated by asterisks.

**Figure 2.**
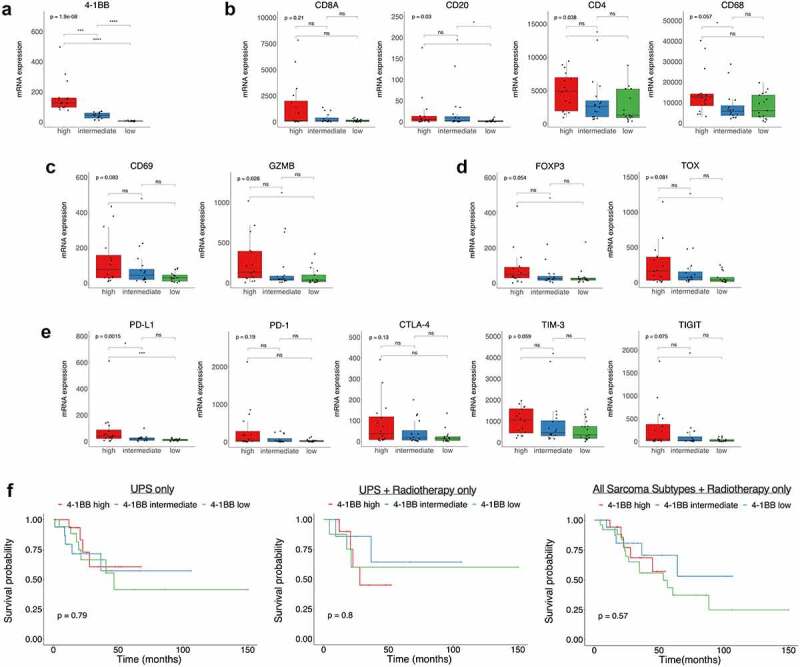
**High 4–1BB (*TNFRSF9*) expression enriches for markers of immune activation and co-inhibitory receptors in UPS TCGA data**. (**a**) A tertile split based on 4–1BB transcript levels was applied to UPS samples in the SARC TCGA dataset. These tertiles are subsequently referred to as 4–1BB-high, -intermediate and -low. For each tertile, mRNA expression was assessed for: (**b**) *CD20, CD68, CD8A* and *CD4* transcripts; (**c**) cytolytic activity via granzyme B (*GZMB*), the activation marker *CD69*; (**d**) the Treg marker *FOXP3* and the T-cell exhaustion marker *TOX*; (**e**) immune co-inhibitory molecules *CD274* (PD-1), *PDCD1* (PD-L1), *CTLA4*, TIM3 (*HAVCR2*) and *TIGIT*. (**f**) Survival probability by 4–1BB subdivision for TCGA data restricted to UPS patients only, UPS patients who received radiotherapy, or all sarcoma patients who received radiotherapy. Significance shown in a-e corresponds to t-test, *p < .05, **p < .01, ***0.001, ****p < .0001.

### MethylCIBERSORT derived hot/cold classification and immune cell composition

Use of MethylCIBERSORT to generate binary ‘immune-hot’ and ‘immune-cold’ subgroups and immune cell population estimates has previously been published and is described in detail.^12^

### Code and data availability

The data accessed in this study are available from The Cancer Genome Atlas Project. The code used for analyses and non-TCGA data are available from the authors upon reasonable request. Code and data availability for immune composition and hot/cold classification has been previously outlined.^12^

## Results

### Immune gene expression differs in patients with UPS compared with MLPS

UPS is considered to be more resistant to radiotherapy.^4^ In the study referenced, 83% of UPS patients tumors had grade 3 disease, with well-differentiated liposarcoma, MLPS, MFS, and LMS ranging from 0 to 36%. The worst 5-year disease-specific survival (DSS) was observed for UPS at 60.1%. In contrast, LMS, MFS, and MLPS were 76.8%, 76.7%, and 84.9%, respectively. 69.2% of UPS patients in that study received radiotherapy, substantially higher that the overall rate of 48.2% across the sarcoma subtypes studied. MLPS has been shown to be radiosensitive relative to other soft tissue sarcomas.^21^ In that study, where all patients received radiotherapy and surgery, the 5-year overall survival for MLPS was 93.9%, compared to 76.4% for all other soft tissue sarcoma subtypes. In this study, we initially compared gene expression between UPS and MLPS in a retrospective cohort of patients receiving radiotherapy and surgery at our institute. This selection was based on the frequency with which subtypes have been shown to be treated at our center with radiotherapy.^4^ We sought to identify potential immunotherapy targets of relevance to UPS patients who progress after surgery and radiotherapy, as well as to improve our understanding of the immune contexture of sarcoma subtypes.

Archival histopathological samples were retrieved from our institution’s tissue bank. These were restricted to patients with a histologically confirmed diagnosis of UPS or MLPS occurring in the extremities who received neoadjuvant radiotherapy prior to surgical resection. A number of MFS samples were included in some but not all analyses. Due to distinct clinical outcomes due to radiotherapy, and potentially differing immunological differences we did not pool UPS and MFS as a molecularly similar spectrum of disease as described in TCGA analyses.^13^ All samples were biopsies taken before the start of therapy. Tumor characteristics of the 27 samples included are outlined in Table 1. UPS patients receiving radiotherapy had a higher rate of progression (Figure 1(a)), the shortest time to progression (Figure 1(b)), and the highest proportion of progression attributed to distant metastasis (Figure 1(c)). These data are in keeping with previous findings from analyses of 556 ESTS patients.^4^ 2/11 UPS patients in our cohort did not receive radiotherapy and are not included in Figure 1(a-c).

**Table 1.** Patient and treatment characteristics

	Entire cohort (n = 27)	MLPS (n = 10)	MFS (n = 6)	UPS (n = 11)
**PATIENT CHARACTERISTICS**				
Age (years)				
Median	62	52	64.5	66
IQR	22.5	20.5	32.75	6.5
Gender				
Male	15	7	2	6
Female	12	3	4	5
Tumor site				
Buttock	4	2	1	1
Thigh	17	8	3	6
Calf	1	0	0	1
Shoulder girdle	5	0	2	3
Grade				
1	4	3	1	0
2	10	4	5	1
3	8	0	0	8
Unknown	5	3	0	1
Tumor depth				
Deep	25	9	6	10
Superficial	2	1	0	1
Adjuvant radiotherapy				
Yes	25	10	6	9
No	2	0	0	2
**TUMOR CHARACTERISTICS**				
Max. tumor diameter (cm)				
Median	9.3	9.6	10.2	8.5
IQR	5.8	4.75	5.3	5.8
Smallest margin (mm)				
Median	1.2	1	2	1.4
IQR	1	1.4	2.025	1
*Clear of margins (n)*	1	1	0	0
Progression				
Local	3	0	2	1
Distant	10	3	1	6
N/A	10	5	2	3
Lost to followup	4	2	1	1

Tumor areas were outlined by an expert pathologist. After macrodissection, samples with sufficient RNA (9/10 MLPS and 7/11 UPS samples passed RNA quality control) were analyzed using the NanoString pan-cancer immune panel with a custom 30-gene probe set (see methods). Transcript analyses were restricted to a comparison between MLPS and UPS, including the two UPS sample that did not receive radiotherapy outlined above. Immune cell populations were assessed using a validated immune-cell score method.^19^ This indicated higher dendritic cell (DC), macrophage, natural killer (NK), and T-cell populations, as well as CD45 overall, in UPS samples compared to MLPS (Figure 1(d)). Differential gene expression indicated 69 genes with a significant difference between UPS and MLPS, shown grouped by nonhierarchical clustering (Figure 1(e)). A number of potential immunotherapy targets were differentially expressed.

As our initial aim focused on identifying immunotherapy targets of relevance to UPS patients who progress after radiotherapy and surgery, of these 69 genes we searched the literature for previous publications on *HAVCR2* (TIM-3), *NT5E* (CD73) and *TNFRSF9* (4–1BB/CD137, referred to from this point as 4–1BB). This was due to immunotherapy agents associated with these genes being under clinical investigation. A search of the literature indicated few studies investigating 4–1BB or other costimulatory molecules, such as OX40, GITR, or ICOS across sarcoma subtypes. Costimulatory signaling is required alongside T-cell receptor (TCR) signaling for full T-cell activation, with agonists against 4–1BB, OX40, GITR, and ICOS under clinical development.^22^ High expression of co-stimulatory molecules has been identified on Tregs, with Treg depleting-antibody-based approaches suggested as, paradoxically, an alternative to agonism.^17^ We focused on the potential of costimulatory molecules across sarcoma subtypes as targets for agents currently under investigation,^15^ and to improve our understanding of the immune contexture of costimulatory molecule expression in sarcoma.

### Analyses of 4-1BB in TCGA data indicate correlation with transcripts for immune activation and co-inhibitory receptors

To corroborate our finding that 4–1BB/*TNFRSF9* mRNA transcripts were higher in UPS (Figure 1), we extended our study to UPS data present in the TCGA dataset. As the analysis of our retrospective cohort (Figure 1) was performed on pre-radiotherapy diagnostic biopsies, 49 available TCGA UPS samples were used in this initial analysis, not just those receiving adjuvant radiotherapy. We first focused on establishing, in this larger dataset, the correlation between 4–1BB transcript levels and markers of an inflamed or exhausted phenotype. This used an even tertile split of UPS samples in the TCGA dataset into high, intermediate, and low groups based on 4–1BB transcript levels (Figure 2(a)).

We looked at *CD8A, CD20, CD4*, and *CD68* in TCGA data for UPS split by 4–1BB status (Figure 2(b)). The highest transcript levels of CD20 and CD4 were observed in the 4–1BB-high group, both showing statistical significance. On an individual sample basis, patients with the highest transcript levels for CD8A fell within both the high and intermediate groups, but no significance was observed between groups. The trend for CD68 was less clear. Looking at transcripts linked to activation, there was a significant difference in *GZMB* and *CD69* (Figure 2(c)). Significant increases were observed in the 4–1BB-high group versus 4–1BB-low for both the Treg marker *FOXP3*, and the T-cell exhaustion marker *TOX* (Figure 2(d)). This corresponded to the highest levels of co-inhibitory receptor transcripts (*PDCD1*/PD-1, *CD274*/PD-L1, CTLA4, *HAVCR2*/TIM3, and *TIGIT*) occurring in the 4–1BB-high group (Figure 2(e)).

To determine if 4–1BB transcript levels were linked to patient outcomes, we assessed survival in 4–1BB-high, intermediate, and low groups for either the 49 UPS patients in the TCGA dataset, only UPS patients receiving adjuvant radiotherapy (25/49), or all sarcoma patients who received radiotherapy (61/235) (Figure 2(f)). Patients were ineligible for inclusion in TCGA sample collection if they had a history of systemic chemotherapy for sarcoma or if their tumor had undergone prior radiotherapy.^13^ 4–1BB was not a prognostic marker in any of these three analyses. This is in keeping with data for PD-L1, where it is not a prognostic marker in NSCLC patients treated with chemotherapy,^23^ but is linked to response rates to PD-1 blockade.^24^

To summarize, 4–1BB is significantly elevated in UPS versus MLPS in our retrospective cohort. Assessment of immune populations in our retrospective cohort, and subsequent TCGA analyses, indicates an inflamed, but exhausted, environment in UPS patients with high 4–1BB levels.

### An analysis of costimulatory molecules indicates OX40 and 4-1BB expression in UPS ranks amongst the highest in the TCGA dataset with OX40 also high in MFS and DDLS

Up until this point we focused on 4–1BB, as we had identified it as differentially expressed in analyses of our retrospective cohort of patient samples (Figure 1). We wished to expand our analyses to other co-stimulatory receptors due to studies showing co-expression, associated with tumor resident Tregs.^17^ Sample numbers may have precluded these from being observed in our original differential expression-based analyses of our retrospective cohort. Therefore, we also investigated the costimulatory receptors OX40, GITR, and ICOS. We were concerned that 4–1BB, whilst high amongst sarcoma subtypes, may not be high relative to other inflamed tumor types. We additionally decided to benchmark sarcoma subtype-specific expression against other cancers in the TCGA dataset to give context to the levels observed in sarcoma.

**Figure 3.**
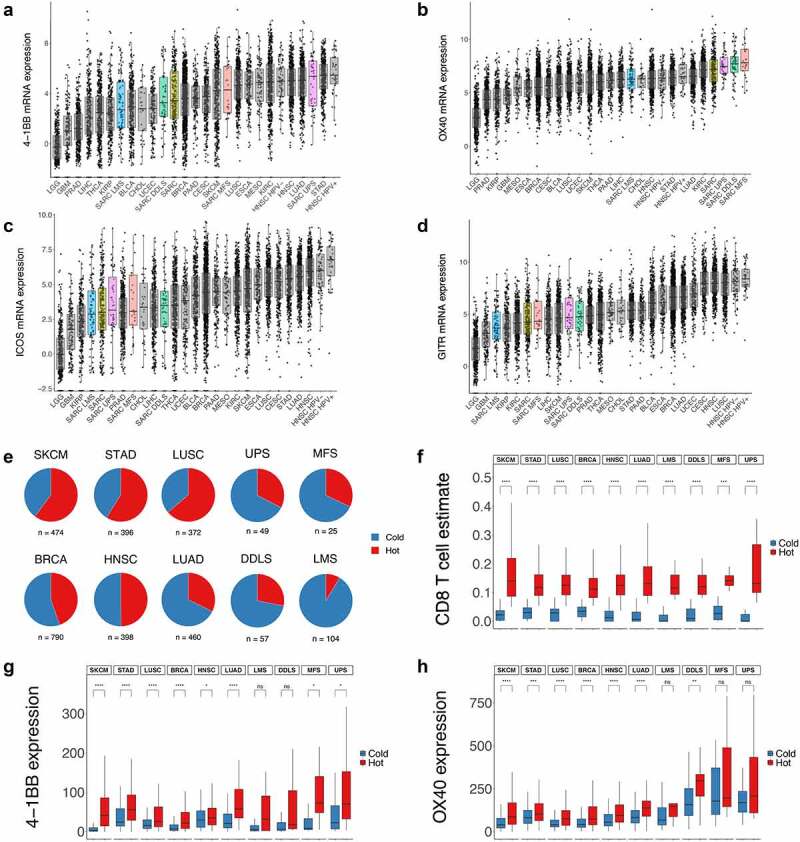
**Analysis of co-stimulatory receptors indicates OX40 mRNA expression in UPS, DDLS and MFS is amongst the highest in the TCGA dataset**. (**a-d**) 4-1BB (*TNFRSF9*), OX40 (*TNFRSF4*), ICOS and GITR (*TNFRSF18*) mRNA expression, split by sarcoma subtype, compared to a range of cancers in the TCGA dataset. (**e**) MethylCIBERSORT-derived binary hot/cold immune classification status comparing sarcoma subtypes to known immune-hot or immunotherapy-responsive cancers in the TCGA dataset. (**f**) A comparison of MethylCIBERSORT-derived CD8 estimates for each sarcoma subtype, split by hot/ cold classification, versus tumours from cancers high in immune-infiltrates or immunotherapy-responsive. (**g-h**) An analysis of the mRNA expression of 4-1BB (*TNFRSF9*) and OX40 (*TNFRSF4*) in sarcoma subtypes and tumours from cancers high in immune-infiltrates or immunotherapy responsive, split by hot/cold classification. All statistical comparisons are between immune-hot and immune-cold as shown, using pairwise wilcox test with Bonferroni correction, *p<0.05, **p<0.01, ***0.001, ****p<0.0001. Abbreviations: BLCA, Bladder Urothelial Carcinoma; BRCA, Breast carcinoma; CESC, Cervical squamous cell carcinoma; CHOL, Cholangiocarcinoma; ESCA, Esophageal carcinoma; GBM, Glioblastoma multiforme; HNSC, Head and Neck squamous cell carcinoma; HPV, Human Papiloma Virus; KIRC, Kidney renal clear cell carcinoma; KIRP, Kidney renal papillary cell carcinoma; LGG, Brain Lower Grade Glioma; LIHC, Liver hepatocellular carcinoma; LUAD, Lung adenocarcinoma; LUSC, Lung squamous cell carcinoma; MESO, Mesothelioma; PAAD, Pancreatic adenocarcinoma; PRAD, Prostate adenocarcinoma; SARC, Sarcoma; SKCM, Skin Cutaneous Melanoma; STAD, Stomach adenocarcinoma; THCA, Thyroid carcinoma; UCEC, Uterine Corpus Endometrial Carcinoma.

Plotted against other cancer types in the TCGA dataset, 4–1BB transcripts in UPS were amongst the highest (Figure 3(a), Supplementary Figure S1a). As non-ligand blocking 4–1BB agonists, such as urelumab, have been shown to promote ligand-dependent receptor clustering,^25^ we also assessed 4–1BBL/*TNFSF9* levels. 4–1BBL transcripts mirrored the findings of 4–1BB, with some of the highest levels detected in UPS and MFS (Supplementary Figure S1). Analysis of the transcript levels of the costimulatory receptors OX40/*TNFRSF4* (Figure 3(b)), ICOS (Figure 3), and GITR/*TNFRSF18* (Figure 3(d)) indicated that ICOS and GITR transcripts were not highly expressed in any sarcoma subtype relative to other cancers in the TCGA dataset. In contrast, OX40 transcript levels in UPS, DDLS, and MFS had the highest expression out of all cancers analyzed. In our retrospective cohort, variability, and sample numbers prevent a definitive statement on which subtype contained the highest levels of OX40 transcripts (Supplementary Figure S2) though the average was highest for UPS. Additionally, OX40 and 4–1BB transcript levels do not simply track with the expression of immune cell infiltrates, *CTLA4, PDCD1*, or *CD274* in TCGA data (Supplementary Figure S3).

We sought to account for the variability in immune infiltration across cancer types by using an independent immune classification approach. This would allow a ‘like-for-like’ comparison between hot sarcoma tumors and hot-tumors from other cancers. We selected melanoma (SKCM), stomach adenocarcinoma (STAD), lung (LUSC, LUAD), breast (BRCA), and head and neck cancer (HNSC) as inflamed or established immunotherapy-responsive cancer types against which to benchmark sarcoma subtypes. These immune hot or immunotherapy responsive cancers can contain high levels of tumor antigens linked to UV exposure (melanoma), tobacco carcinogens (lung), DNA-damage response defects (breast), or viral antigens (HPV-positive head and neck cancer). This is in contrast to sarcoma, where no major genetic defect or environmental mutagen has been identified that correlates to higher levels of tumor antigens. We used a published method of binary classification (immune-hot or immune-cold) based on MethylCIBERSORT-derived estimates of cell abundances in mixed tumor populations.^12^ This binary hot/cold classification indicated UPS, MFS, and DDLS possessed similar levels of immune-hot tumors, with LMS substantially colder (Figure 3(e)). Sample numbers per subtype classified as hot/cold were: UPS, 16/33; MFS, 8/17; DDLS, 16/41; LMS 9/95. Immune-hot sarcomas were less frequent than other inflamed cancer types (Figure 3(e)), but still comprised approximately one-quarter to one-third of UPS, MFS, and DDLS tumors. These findings are consistent with a recent study on sarcoma immune classes.^11^ In our ‘like-for-like’ comparison, the increase in CD8 estimates for tumors classified as hot versus cold was highly similar between all sarcoma subtypes and inflamed or immunotherapy-responsive cancers (Figure 3(f)). Sarcoma subtypes were also broadly similar to immunotherapy responsive cancers in regard to the trends observed for Treg, CD4-effector, B cell, and NK cell estimates between hot and cold tumors (Supplementary Figure S4).

We then asked the question, if there were differences between 4–1BB and OX40 transcript levels across the cancers selected, when split by hot/cold classification. 4–1BB transcripts were higher in immune-hot tumors compared to immune-cold tumors for sarcoma subtypes and the other cancers analyzed (Figure 3(g)). While hot-UPS tumors had the highest average 4–1BB transcript levels, hot-LUAD, hot-STAD, and hot-SKCM also had high levels. For OX40 (Figure 3(h)), the majority of cancers showed higher OX40 transcripts in tumors classified as immune-hot versus those classified as immune-cold. This pattern diverged for UPS and MFS. This appeared to be due to higher levels of OX40 transcripts in tumors classified as immune-cold. OX40 transcripts were higher in UPS, MFS, and DDLS verses other cancers for both hot and cold tumors. Statistical comparisons of UPS, MFS, and UPS versus other cancers confirmed this difference (Supplementary Table 1).

To summarize, expression levels of 4–1BB in UPS and OX40 in UPS, MFS, and DDLS are amongst the highest in the TCGA dataset. Immune-hot sarcomas across all subtypes have similar estimated levels of CD8 T-cells compared to established immunotherapy-responsive cancers. Hot-UPS tumors have some of the highest levels of 4–1BB, not only when compared with other sarcoma subtypes but also compared with hot-tumors from immunotherapy-responsive cancer types. OX40 transcripts were higher in UPS, MFS, and DDLS compared to other cancer types. The high levels of OX40 observed in UPS, MFS, and DDLS are due to increases in tumors classified as both hot and cold.

### OX40 and 4-1BB delineate distinct immune profiles

**Figure 4.**
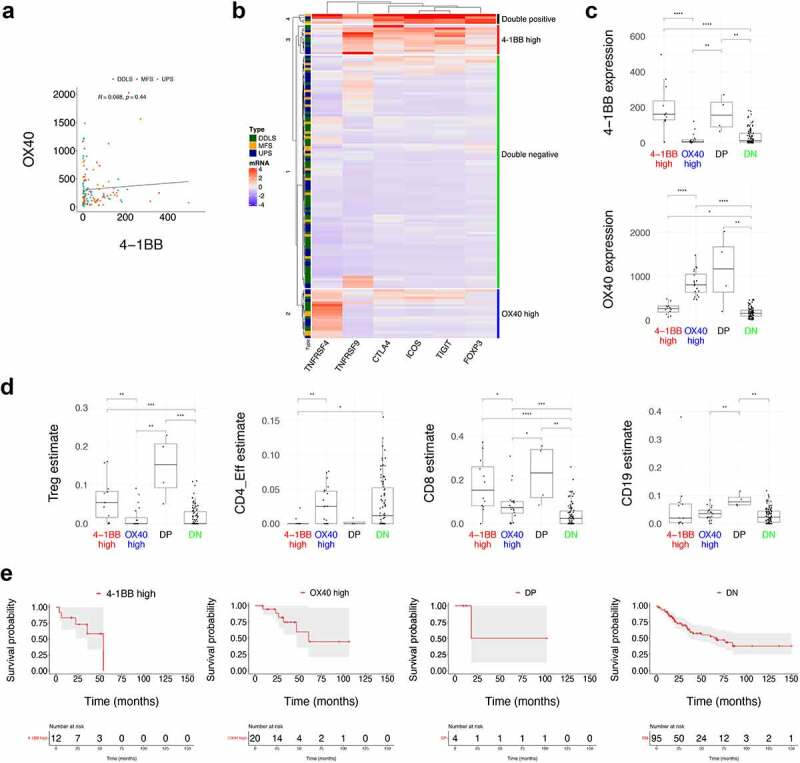
**OX40 and 4-1BB delineate distinct immune profiles**. (**a**) Correlation analysis was performed between OX40 (*TNFRSF4*) and 4-1BB (*TNFRSF9*) transcripts in TCGA data restricted to UPS, MFS and DDLS sarcoma subtypes indicating no clear correlation. (**b**) K-means clustering was performed using TCGA data restricted to UPS, MFS and DDLS sarcoma subtypes for OX40 transcripts, 4-1BB transcripts and other transcripts associated with tumour-resident Tregs. The four clusters identified were labelled OX40-high, 4-1BB-high, double negative, and double positive. (**c**) 4-1BB and OX40 mRNA expression is shown for each of the four clusters shown in b. Abbreviations: DP, double positive for 4-1BB and OX40; DN, double negative for 4-1BB and OX40. (**d**) MethylCIBERSORT-derived immune population estimates corresponding to each of the four clusters in panel b. (**e**) Survival probability for each cluster identified in panel b plotted individually with grey areas indicating 95% confidence intervals. Statistical analysis shown in all panels is by Wilcoxon test between the groups indicated, *p<0.05, **p<0.01, ***0.001, ****p<0.0001.

Having identified OX40 and 4–1BB as potential immunotherapy targets of relevance to UPS patients who fail to respond to radiotherapy or surgery, we wished to further profile their expression to increase our understanding of the immune contexture of sarcoma. Recent research has shown OX40 and 4–1BB are both highly expressed on tumor-associated Tregs.^17^ Our initial expectation was that the high expression of OX40 and 4–1BB in the TCGA analysis (Figure 3) was indicative of a singular Treg-rich phenotype. However, the differences observed, when split based on a hot and cold tumor classifier (Figure 3(g,h)), suggested this may not be the case. While 4–1BB expression was generally statistically different between hot and cold classifications, this was not the case in three of four sarcoma subtypes for OX40. Due to the low levels of hot-tumors in LMS, we focused our subsequent analyses on DDLS, MFS, and UPS (131 samples in total). We plotted 4–1BB transcripts against OX40 transcripts and were surprised to find no clear correlation between the expression levels (Figure 4(a)).

Alongside *TNFRSF4*/OX40 and *TNFRSF9*/4-1BB, we selected a number of markers of tumor-resident Tregs (*CTLA4, ICOS, TIGIT, and FOXP3*) and performed k-means clustering for all UPS, MFS, and DDLS samples in the TCGA (Figure 4(b)). *TNFRSF9*/4-1BB transcript levels closely aligned with the pattern of expression observed for *CTLA4, ICOS, TIGIT*, and *FOXP3*. However, *TNFRSF4*/OX40 delineated separate immune profiles compared to the other transcripts included. K-means clustering indicated four distinct profiles. The first cluster contained low levels of all transcripts (double negative). The second cluster contained high levels of *TNFRSF4*/OX40 only (OX40 high). The third cluster contained high levels of all other transcripts except *TNFRSF4*/OX40 (4–1BB high). The fourth cluster contained high levels of all transcripts (double positive for *TNFRSF4*/OX40 and *TNFRSF9*/4-1BB). Plots of absolute levels of *TNFRSF4*/OX40 and *TNFRSF9*/4-1BB based on the k-means clustering-derived groups indicated the clear disparity in transcript levels between each group (Figure 4(c)). We repeated the clustering approach on data for LUAD, HPV+ HNSCC, and HPV-HNSCC. We observed the same pattern of expression for each cluster (Supplementary Figure S5), indicating that the immune profiles identified are not unique to sarcoma.

We plotted MethylCIBERSORT-derived population estimates for each cluster to determine how *TNFRSF4*/OX40 and *TNFRSF9*/4-1BB levels correlated to specific immune populations (Figure 4(d)). The clearest differences were in CD8, CD19, CD4-effector, and Treg population estimates. The double positive cluster corresponded to the highest estimates for CD19 and Treg populations. Clusters with high 4–1BB (double positive or 4–1BB high) contained high levels of CD8 and Treg estimates, but very low CD4-effector cell estimates. The OX40 high only cluster contained an intermediate level of CD8 estimates. We plotted survival probabilities (Figure 4(e)) corresponding to each of the four clusters identified in Figure 4(b). Due to small numbers, resulting in large confidence intervals for three of the four clusters, we could not clearly observe any differences in overall survival.

In summary, *TNFRSF4*/OX40 and *TNFRSF9*/4-1BB transcript levels can delineate distinct immune profiles with unique inferred immune populations present within each.

### OX40+ Tregs can be TLS-associated or TLS-independent

We wanted to gain a better understanding of the populations on which OX40 can be highly expressed (Figure 3) and if we could potentially identify the non-Treg-associated populations that may be responsible for the OX40-high only cluster in Figure 4. We performed multiplex immunohistochemistry on our retrospective pre-treatment biopsies (Figure 1) to try to answer these questions. We restricted our analyses to UPS and MFS, due to the low number of immune cells detected in MLPS (Figure 1(d)). 8/11 UPS and 5/6 MFS samples passed IHC quality control and were retained for downstream analyses.

**Figure 5.**
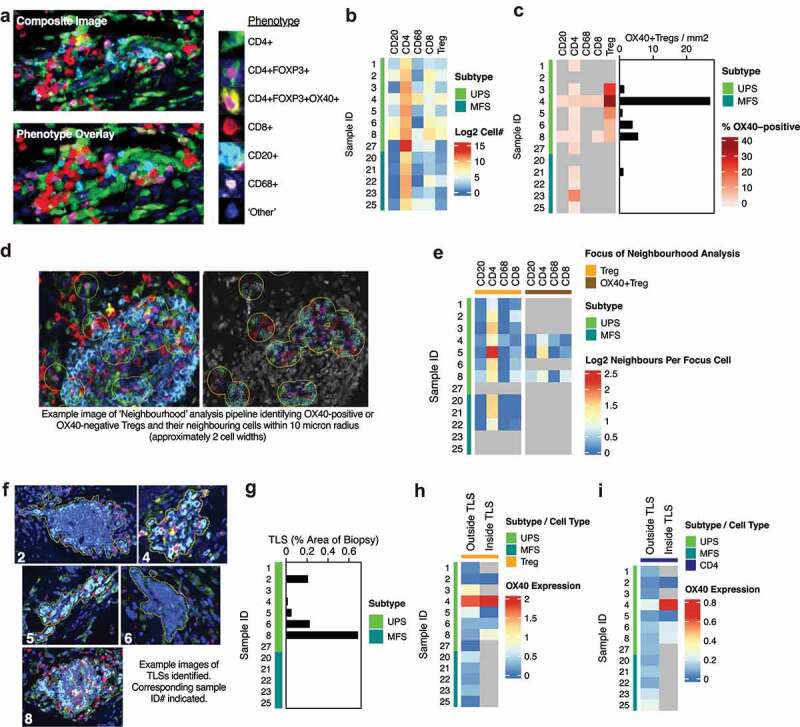
**OX40+ Tregs exhibit both a TLS-associated and TLS-independent phenotype in UPS**. Pre-treatment UPS and MFS biopsies were analyzed by multiplex immunohistochemistry. (**a**) Slides were stained for CD4, FOXP3, OX40, CD8, CD20, and CD68, with DAPI as nuclear stain. Example images and phenotype classification are shown. (**b**) Immune cell populations were quantified using QuPath. Log2 values for each cell population per mm^2^ are shown corresponding to UPS and MFS biopsies as indicated. (**c**) OX40 staining on each cell was classified as positive or negative. The number of OX40-positive cells is expressed as a percentage of each parent population. Grey indicates less than ten OX40-positive cells were identified for a given parent population, and were excluded due to low numbers. As OX40 strongly associated with Tregs, the number of OX40-positive Tregs per mm^2^ is also shown. (**d-e**) Neighborhood analysis was performed on OX40-positive Tregs and OX40- negative Tregs (referred to as Treg only). An overview of the image pipeline is described. Immune cells quantified to be approximately two cell widths from OX40-positive Tregs or OX40-negative Tregs are shown. Grey indicates insufficient cell numbers to perform neighborhood analysis. (**f-i**) OX40 expression on Tregs and CD4s was performed assessing differences between immune cells that are TLS-associated, or cells in surrounding tissue areas classified as TLS-independent. Example images of TLSs identified are shown (f) along with the percentage area identified as a TLS relative to the total biopsy tissue area (g). (h-i) OX40 expression on CD4+ FOXP3+ Tregs and CD4+ FOXP3- CD4s was assessed based on their location outside TLSs (TLS-independent) or inside TLSs (TLS-associated).

Example images of multiplex staining for CD20, CD68, CD4, CD8, FOXP3, and OX40 are shown, along with phenotype identification (Figure 5(a)). We observed a clear pattern of higher numbers of CD20, CD8, and CD4+ FOXP3+ (Tregs) in UPS compared to MFS (Figure 5(b)). OX40 staining intensity on each immune cell population was scored as positive (OX40+) or negative (OX40-). This indicated that OX40 was highly expressed on Tregs in UPS samples (Figure 5(c)). Our expectation was that we would find OX40+ Tregs, but potentially also identify the population of immune cells expressing OX40 in the OX40-high only cluster that did not contain Treg-associated transcripts (Figure 4(b)). We found evidence of OX40 expression on CD4+ FOXP3- (CD4) cells (Figure 5(c)). Unlike CD4+ OX40+ FOXP3+ cells, CD4+ OX40+ FOXP3- cells comprised a much lower percentage of the total CD4+ FOXP3- cell population. Despite the large number of CD8, CD20, and CD68 cells present in some UPS samples, there was little evidence pointing to OX40 expression on these cell populations (Figure 5(c)). This suggests CD4+ FOXP3- cells are the most probable source of the OX40-high only cluster identified previously (Figure 4(b)). This is consistent with the high levels of CD4 effector cells seen in this cluster (Figure 4(d)).

One UPS sample had a substantial number of OX40-positive Tregs, with one-third of all Tregs in this sample (sample ID 4) being classified as OX40-positive (Figure 5(c)). We were interested in the spatial profile of these OX40-positive Tregs in comparison to OX40-negative Tregs. Therefore, we performed a ‘neighbourhood’ analysis of cells within approximately two cell widths (Figure 5(d,e)). There was no definite sign of differences between the proximity of cells to OX40-negative or OX40-positive cells (Figure 5(e)). There was an indication that there may be a decrease in CD4-effector cells in the neighborhood of OX40-positive Tregs. UPS sample 6 was unusual, in that despite containing similar levels of OX40-positive Tregs to UPS sample 8, these cells were remote from other immune cells. Therefore, neighborhood analysis was not possible (Figure 5(e)).

During neighborhood analysis, we observed the presence of TLSs. TLSs are lymphoid formations that form within non-lymphoid tissue and are classified as aggregates of B-cells arranged in a follicular structure, surrounded by a region of T-cells.^11^ Many of these were early-TLSs containing lymphocytic aggregates of B and T-cells without higher order structures, or primary follicle-like TLSs that are large in size and contain centralizing B-cells. In the case of sample 8, these were more mature secondary follicle-like TLSs. We asked if there was a clear spatial difference between OX40 levels on Tregs and CD4 cells based on their location inside or outside TLSs. Example images of identified TLSs are shown (Figure 5(f)) alongside a quantification of the total biopsy area occupied by TLSs (Figure 5(g)). UPS samples 4 and 8 were the two most interesting due to possessing the highest numbers of Tregs aiding robust analysis. Sample 4 contained minimal TLSs, but a substantial number of Tregs. Sample 8 contained the second highest number of Tregs, the highest area occupied by TLSs, and TLSs exhibiting the greatest maturity. Tregs associated with TLSs in sample 8 had OX40 expression 4-fold higher than Tregs outside TLSs (Figure 5(h)). OX40 expression on Tregs both inside and outside TLSs was high, but similar, for sample 4. In three out of five samples, OX40 expression on CD4 cells inside TLSs was double that of CD4 cells outside TLSs (Figure 5(i)).

While the sample numbers associated with these observations are low, they give two highly distinct OX40 Treg phenotypes in UPS. One where OX40 expression is high on Tregs that are diffusely spread through the tumor, independent of TLSs. The other has TLS-associated Tregs that have substantially higher OX40 expression than the diffusely spread, TLS-independent Treg population.

## Discussion

UPS patients have poor outcomes in comparison to other sarcoma patients treated with radiotherapy and surgery.^4,21^ In this patient population with a clinically unmet need, our study supports the investigation of immunotherapy agents targeting the costimulatory molecules 4–1BB and OX40. Our analyses indicate differing immune profiles linked to co-stimulatory molecule expression in sarcoma, with further variation observed in the spatial profile of OX40+ Tregs in relation to tertiary lymphoid structures.

Despite a higher probability of elevated immune infiltration, UPS patients have poorer responses to radiotherapy and surgery. Data from the SARC028 trial have shown the encouraging activity of pembrolizumab in patients with UPS and DDLS, but poor response rates in synovial sarcoma, leiomyosarcoma, and osteosarcoma.^7,11,14^ The use of immunotherapy in sarcoma patients is beset with the same issues that arise with other tumor types, namely an absence of useful predictive markers for patient stratification and lack of clarity on which alternative options to use in those refractory to anti-PD-1- and/or anti-CTLA-4-based regimens.

Unlike in non-small lung cancer,^26^ we found no evidence that OX40 or 4–1BB was a favorable prognostic factor in sarcoma. High transcript levels of 4–1BB and OX40 in UPS, MFS, and DDLS, indicate that these sarcoma subtypes are likely to be a promising cohort of patients for future immunotherapy agents targeting these pathways.^27^ With emerging data on pembrolizumab from the SARC028 trial, this is likely to be in combination with anti-PD-1 axis blockade. Other tumor types have benefitted from dual-immunotherapy combinations, such as anti-PD-1 and anti-CTLA-4. Data from the Alliance A091401 study of nivolumab and ipilimumab suggests this may also be true for sarcoma.^8^ In a primary versus transplant preclinical mouse model of UPS, primary tumors were resistant to radiotherapy and dual-checkpoint blockade with PD-1 and CTLA-4.^28^ In that study, single-cell sequencing indicated low numbers of activated T cells in primary tumors. Targeting co-stimulatory molecules such as OX40 and 4–1BB may represent an approach to overcome this immune tolerance.^16,17^

With the success of the SARC-028 trial of pembrolizumab, and with few sarcoma-specific immune-profiling studies in the literature,^13,29^ there is a need to increase our understanding of the immune contexture of sarcoma. To our knowledge, immune profiles linked to differing expression patterns of co-stimulatory molecules have not previously been identified. A prior publication looking only at tumor resident Tregs suggested a homogeneous expression of co-stimulatory molecules on Tregs.^17^ Our use of a binary hot-cold classification method is consistent with other studies,^11,13^ with hot-tumors found distributed across UPS, MFS, and DDLS, with immune-hot LMS tumors much less frequent. Although patients with LMS might benefit from immunotherapy, these data suggest that their inclusion in immunotherapy trials should be restricted to those with immune-hot tumors. In regard to CD8, CD4, and Treg estimates, hot-tumors across all four sarcoma subtypes were broadly similar to hot-tumors in immunotherapy-responsive cancers. Hot-LMS, whilst significantly less prevalent than hot-tumors in UPS, MFS, or DDLS, is not an outlier in regard to the pattern of immune infiltration observed. Even for UPS, only a third of patients are immune-hot (Figure 3), with different immune profiles (Figure 4), and with distinct spatial features identifiable for OX40+ Tregs (Figure 5). This indicates how key biomarker discovery is likely to be in appropriately selecting UPS patients for future immunotherapy trials. The dual high population of 4–1BB and OX40 highlights a profile of sarcoma patients with the most inflamed tumors. These patients may potentially have a favorable response to immunotherapies, either anti-PD-1 alone or in combination with novels agents targeting 4–1BB or OX40.

Two major recent publications in the field have been translational studies on the SARC028 trial of pembrolizumab, focused on UPS and DDLS. Baseline tumor biopsies indicated responders had higher infiltration of T lymphocytes and a higher percentage of PD-L1^+^ macrophages, as well as effector memory and regulatory T cells.^14^ Petitprez et al. carried out analyses across a number of STS cohorts, identifying five sarcoma immune classes with histological subtypes evenly distributed across the most immune-rich classes. Patients in the SARC028 trial with high B cells and tertiary lymphoid structures (TLSs) exhibited the highest objective response rate to PD-1 blockade.^11^ Our findings indicate tumors containing TLSs can be further differentiated by the spatial location of OX40+ Treg. These profiles offer avenues of investigation to determine if these signatures are linked to response rates in future immunotherapy trials in sarcoma. Of particular interest would be the SU2C-SARC-032 trial of pembrolizumab and radiotherapy in UPS and DDLS, particularly in light of preclinical data indicating the possible deleterious effects of radiotherapy on non-tumor lymphoid tissue.^30,31^ With the identification of TLSs in sarcoma and the link to responsiveness to pembrolizumab,^11^ clinical data on the impact on TLSs in radiotherapy plus ICI trials would be highly informative.

As stated previously, TLSs have been shown to associate with improved survival in response to immunotherapies.^11,32^ Our data on OX40+ Tregs and OX40+ CD4s, as TLS-associated or TLS-independent, provides further insight into the complexity of the immune contexture of TLSs in sarcoma. OX40 is known to be expressed most highly on Tregs, yet MethylCIBERSORT data for the OX40-high/4-1BB-low cluster indicates low Tregs, but high levels of CD4 effector cells. Our data in Figure 5 indicates that OX40 expression is most likely to be expressed on Tregs and CD4 effector cells. A study in head and neck cancer of OX40 expression on tumor infiltrating T-cells compared to those in the blood indicated that, while OX40 was more highly expressed on tumor resident Tregs, it was elevated to a lesser extent on tumor infiltrating conventional CD4s.^33^ Preclinical studies have shown depletion of TLS-associated Tregs can increase rates of proliferation of CD4+ and CD8 + T-cells in TLSs 5-fold and 10-fold, respectively.^34^ OX40 antibodies, targeted to deplete TLS-associated OX40+ Tregs, could be an approach to expand response rates to anti-PD-(L)1 therapies. Whilst we recognize the small sample numbers, the contrast between the two TLS-independent and TLS-associated OX40+ Treg phenotypes we observed was stark. It would be highly interesting, in future samples from immunotherapy trials in sarcoma, to determine the prevalence of each OX40+ Treg phenotype and if they are predictive for response rates in TLS-positive patients.

There are a number of limitations to our study. These are centered around sample numbers for individual subtypes and concerns around the representative nature of a single biopsy in comparison to the heterogeneity of the tumor mass as a whole. For example, the extent of TLSs detected could be significantly impacted by sampling. The ability to accurately determine differences in overall survival between the 4–1BB and OX40 immunological groups we define were also impacted by low sample numbers. We decided to avoid pooling sarcoma subtypes, an approach used in some publications.^12,13^ While resulting in lower sample numbers, keeping sarcoma subtypes separate has strengths. Classification in the soft tissue sarcoma TCGA publication of UPS and MFS as a single spectrum of disease^13^ contrasts with the disparity observed in our analyses of TLSs and OX40+ Tregs (Figure 5). Beyond the differences in patient outcomes in response to surgery and radiotherapy,^4,21^ the immunological differences identified in our study suggest UPS and MFS should be analyzed separately.

In summary, our data point to a group of immune-hot sarcoma patients that may be highly amenable to OX40-targeted agents. Taken in totality with recent publications on the SARC028 study, these data indicate many more sarcoma patients could benefit from immunotherapy, if given as part of a rationally targeted approach. Our findings around TLSs and OX40+ Tregs provide new insight into the immune contexture of sarcoma, and describe further metrics by which patients could be stratified in analyses seeking to predict responders to immunotherapies.

## Supplementary Material

Supplemental Material
