# Supplementary material for: OX40 and 4-1BB delineate distinct immune profiles in sarcoma
Source: Oncoimmunology. 2022 May 9;11(1):2066050. doi: 10.1080/2162402X.2022.2066050 (PMC9090286; doi:10.1080/2162402X.2022.2066050)
Supplement: Supplemental Material [file KONI_A_2066050_SM0936.docx]

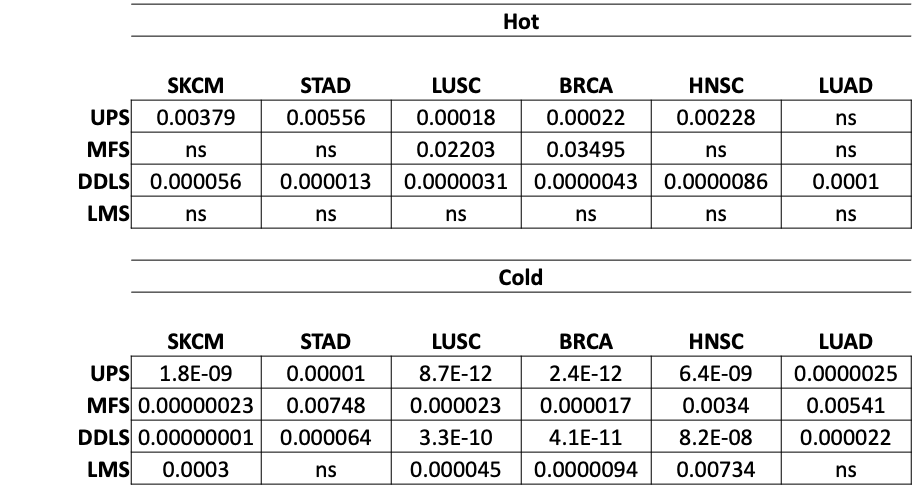


**Supplementary Table 1: Statistical comparison between OX40 (*TNFRSF4*) mRNA expression in TCGA data between sarcoma subtypes and selected cancers.** Statistical comparison of OX40 mRNA expression was performed after splitting into groups based on hot/cold tumour classification (Figure 3h). P-values calculated by pairwise comparison performed using Wilcoxon rank sum test with Bonferroni correction for multiple comparisons.


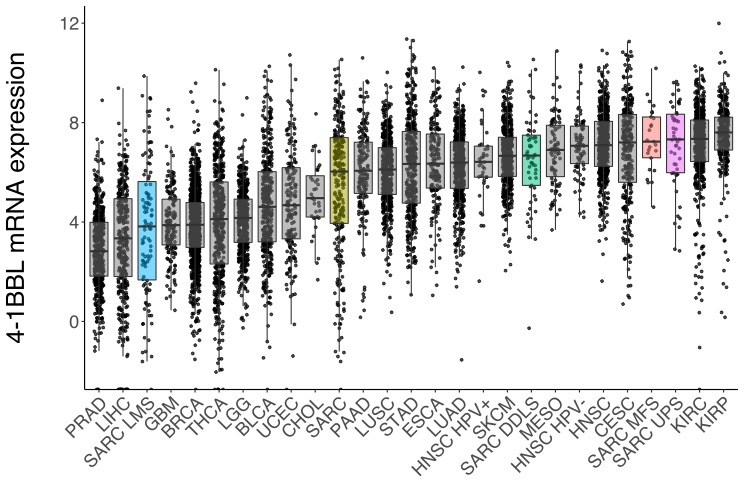


**Supplementary Figure S2. OX40 (*TNFRSF4*) mRNA expression in NanoString data corresponding to Figure 1.** Samples with sufficient RNA were analysed using the NanoString pan-cancer immune panel. Normalised data for MLPS, MFS and UPS indicates OX40 mRNA expression is highest in UPS but was not statistically significant versus MLPS or MFS. Statistical analysis by t-test.

**Supplementary Figure S1. 4-1BB (*TNFRSF9*) and 4-1BBL (*TNFSF9*) expression across sarcoma subtypes in TCGA data. (a-b)** mRNA expression of 4-1BB and 4-1BBL was assessed between sarcoma subtypes indicating higher expression in UPS. **(c)** Expression of 4-1BBL is high in UPS and MFS relative to other cancers in the TCGA dataset. Statistical analysis by Wilcoxon test *p<0.05, **p<0.01, ***0.001, ****p<0.0001.

**A**

**B**

**C**

**Supplementary Figure S3. TCGA expression data for CD45 (*PTPRC*) and *CD8A* transcripts and current clinical immunotherapy targets.** Expression data for sarcoma and split sarcoma subtypes compared to selected other cancer types in the TCGA dataset. (**a**) CD45 (*PTPRC*) and *CD8A*, (**b**) *CD274* (PD-L1), *PDCD1* (PD-1) and *CTLA4*.

**Supplementary Figure S4. MethylCIBERSORT-derived population estimates for additional immune cell populations.** Immune population estimates are displayed as shown in figure 3f for (**a)** Tregs, **(b)** B cells, **(c)** NK cells, and **(d)** CD4 effector cells. Comparison between sarcoma subtypes and immunotherapy responsive cancers. Split by hot (red) and cold (blue) MethylCIBERSORT-derived binary immune classification status. Statistical comparisons in a-b are between hot-UPS or cold-UPS and the corresponding hot (red asterisks) or cold group (blue asterisks) as indicated. Pairwise Wilcoxon test with Bonferroni correction, *p<0.05, **p<0.01, ***0.001, ****p<0.0001.

**A**

**B**

**C**

**D**

**Supplementary Figure S5. OX40 (*TNFRSF4*) and 4-1BB (*TNFRSF9*) also delineate distinct immune profiles in LUAD and HNSCC.** K-means clustering was performed using TCGA data for LUAD, HPV-negative HNSCC and HPV-positive HNSCC for *TNFRSF4*, *TNFRSF9* and other transcripts associated with tumour-resident Tregs. The four clusters indicate a similar expression profile of OX40-high, 4-1BB-high, double negative, and double positive as observed for sarcoma.
